# Transient, recurrent, white matter lesions in x-linked Charcot-Marie-tooth disease with novel mutation of gap junction protein beta 1 gene in China: a case report

**DOI:** 10.1186/s12883-014-0156-5

**Published:** 2014-08-03

**Authors:** Yuan Zhao, Yanchen Xie, Xiaoquan Zhu, Huigang Wang, Yao Li, Jimei Li

**Affiliations:** 1 Department of Neurology, Beijing Friendship Hospital, Capital Medical University, Beijing 100050, People’s Republic of China; 2 Laboratory for Medical Genetics, Institute of Geriatrics, Beijing Hospital, Ministry of Health, Beijing 100730, People’s Republic of China

**Keywords:** Charcot-Marie-Tooth disease, White Matter, Gap junction protein beta l, Connexins, Mutation

## Abstract

**Background:**

Transient white matter lesions have been rarely reported in X-linked Charcot-Marie-Tooth disease type 1.

**Case presentation:**

We describe a 15-year-old boy who presented transient and recurrent weakness of the limbs for 5 days. His mother, his mother’s mother and his mother’s sister presented pes cavus. MRI and electrophysiology were performed in the proband. Gap junction protein beta l gene was analyzed by PCR-sequencing in the proband and his parents. The electrophysiological studies showed a mixed demyelinating and axonal sensorimotor neuropathy. MRI showed white matter lesions in the internal capsule, corpus callosum and periventricular areas, which showed almost complete resolution after two months. T278G mutation in Gap junction protein beta l gene was detected in the proband and his mother.

**Conclusion:**

This case report highlights that the novel T278G mutation of Gap junction protein beta l maybe could result in X-linked Charcot-Marie-Tooth disease type 1 with predominant leucoencephalopathy. The white matter changes in MRI of X-linked Charcot-Marie-Tooth disease type 1 patient are reversible.

## Background

Charcot-Marie-Tooth disease (CMT) is the most frequent hereditary neurological disorder in the world, which affects 1 in 2,500 people. Most CMT diseases are autosomal dominant, the rest are recessive and X-linked forms. CMTX is divided into a dominant CMTX1 and four recessive CMTX2–5. CMTX accounts for approximately 10%-20% of all hereditary demyelinating neuropathies [1].

CMTX is characterized by distal muscle weakness and atrophy with characteristic steppage gait,pes cavus, decreased deep tendon reflexes and sensory loss. Recently, transient white matter lesions in CMTX have been reported, and become a prominent clinical manifestation. CMTX is caused by mutation in the GJB1 gene on chromosome Xq13.1 that codes for the connexin 32 protein (Cx32). Cx32 is expressed in the myelinating Schwann cells of the peripheral nerves. However, the protein is also widely expressed in the oligodendrocyte of the central nervous system (CNS). Cx32 maintains the permeability of myelinating cells [2]. CX32 plays an important an role in the biology of myelin-forming cells Cx32 maintains the permeability of neurons. We analyse a CMTX male patient by the clinical manifestations, imagings and genetic analyses, who developed transient CNS dysfunction accompanied by a Cx32 mutation and diffuse white matter abnormalities.

## Case presentation

A 15-year-old male of Han nationality presented with a sudden weakness of limbs. Five days before being admitted to our hospital, about five pm after school, the patient felt weakness of the lower limbs and walked unsteadily. However, he did not show weakness of both upper limbs, chest congestion, palpitations, dizziness, headache, numbness of the limbs, convulsion and incontinence. About five thirty pm, weakness of his both lower limbs disappeared. But he showed weakness of both upper limbs twice. Three days before admission, he did not write well with his right hand when he was at school. Two days before admission, he could not lift his left arm and had slurred speech, dysphagia, which lasted 2–3 hours before being recovered. On the day of admission, he presented right hemiplegia and numbness. History of past illness showed that he had a high fever two weeks before onset, the highest temperature was 39.9°C, and he was poor in spirit, but did not show cough, expectoration, sore throat or other symptoms. He was treated by Ceftriaxone sodium and dexamethasone, and he got well soon. Two months before onset, he had a history of lower extremity weakness lasting about 30 minutes. He was the product of a normal full-term pregnancy and delivery. He had no learning difficulties. His mother, his mother’s mother and his mother’s sister presented pes cavus.

His medical examination presented the atrophy of his interosseous muscles and his distal lower extremities, flat thenar muscles, poor flexibility of his hands, diminished deep tendon reflexes in all extremities, negative bilateral Babinski signs, positive bilateral Pussep signs and equinovarus. The internal medicine examination showed no abnormalities.

**Figure 1:**
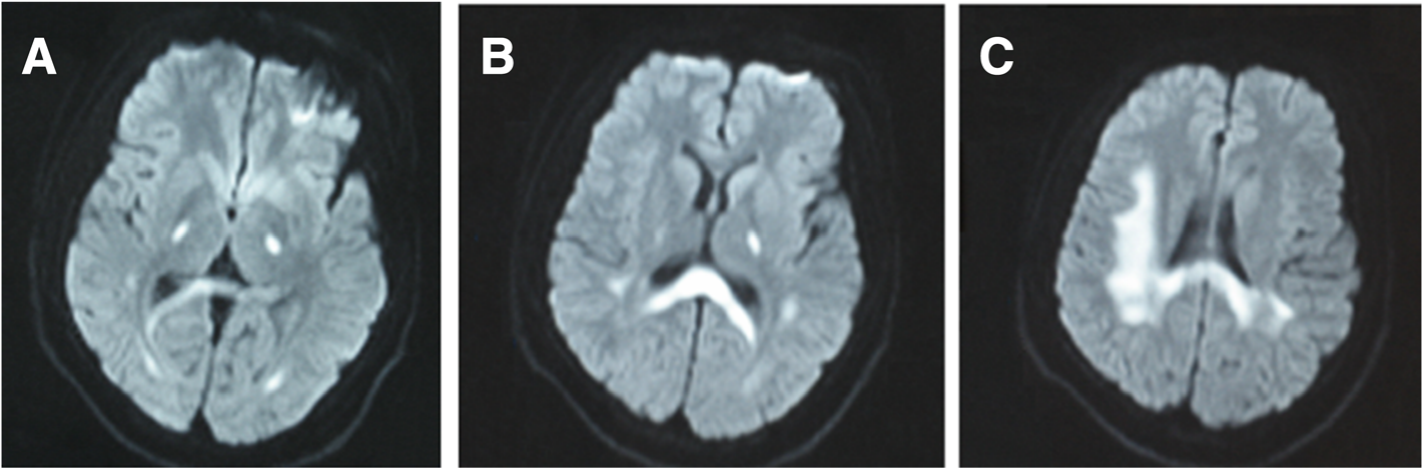
**The first MRI after four days of onset.** Diffusion-weighted MRI after four days of the onset of symptoms, which presents repeated transient weakness of the limbs, slurred speech and dysphagia, shows diffuse signal abnormalities in the internal capsule **(A and B)**, corpus callosum **(B)** and periventricular areas **(C)**. Neurologic examination reveals musclar atrophy of distal limbs, diminished deep tendon reflexes in all extremities and giving the characteristic inverted champagne bottle appearance.

**Figure 2:**
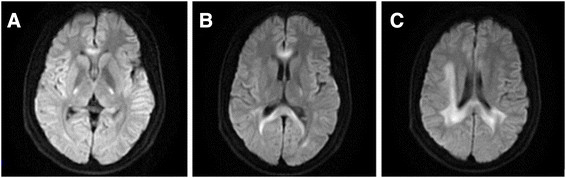
**The second MRI after nine days of onset.** Diffusion-weighted MRI afternine days of the onset of symptoms shows no change compared with the first MRI **(A, B and C)**.

**Figure 3:**
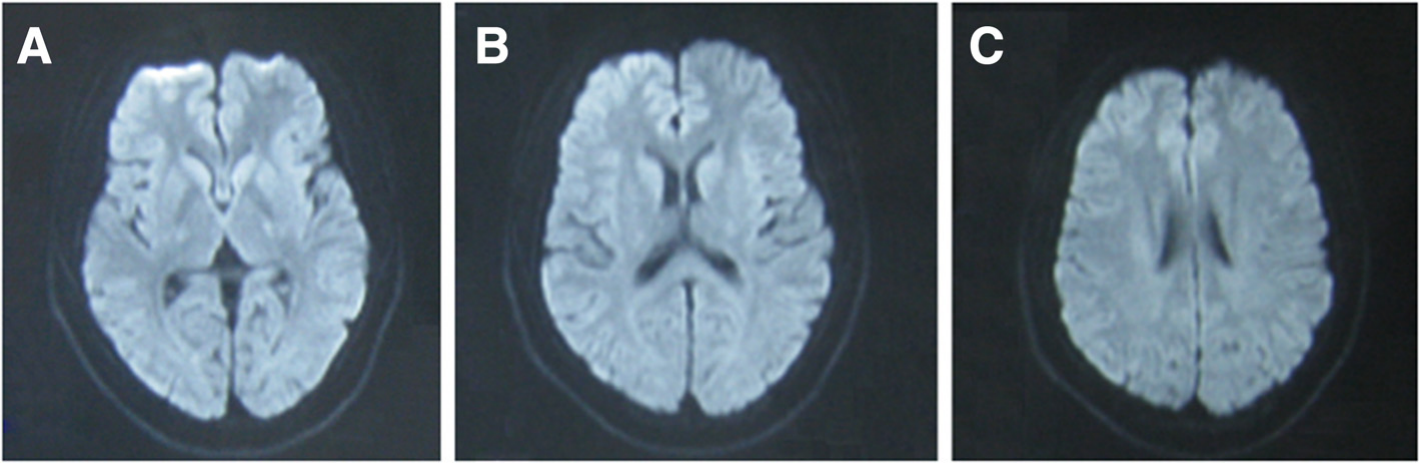
**The third MRI after two mouths of onset.** Diffusion-weighted MRI after two months of onset by which time the patient has no repeated neurological attracks, shows nearly complete resolution of the white matter changes **(A, B and C)**.

His investigations showed normal blood counts and inflammatory markers, renal, liver, electrolytes, sedimentation rate, blood glucose and thyroid function test. Cerebrospinal fluid analysis showed mildly elevated white blood cell (6 × 10^6^/L) and mildly elevated protein (65 mg/dl) without oligoclonal bands. The first MRI after four days of onset of symptoms (Figure 1) revealed diffuse signal abnormalities in the internal capsule, corpus callosum, periventricular areas. The second MRI after nine days of onset (Figure 2) showed no change compared with the first one. The third MRI after two months (Figure 3) showed almost complete resolution of the white matter changes.

Electromyogram presented peripheral neurogenic damages of upper and lower extremities (Table 1). BAEP showed prolongation of latency in bilateral wave III and wave I-III interpeak, poor reproducibility in Wave V graphics. Somatosensory evoked potential of upper limbs presented prolongation of latency in bilateral cortical potential N20 and bilateral subcortical potential N9, N13. Somatosensory evoked potential of lower limbs revealed prolongation of latency on the right side of the cortical potential P40, and that graphic differentiation is not clear on the left cortical potential P40 and bilateral subcortical potential N21.

**Table 1:** Nerve conduction studies

**Nerve**	**Stimulation site**	**Distal latency (ms)**	**Amplitude (sensory in μV, motor in mV)**	**Velocity (m/s)**
Sensory				
Left median	Finger I		3.2 (↓92%)	33 (↓42%)
	Wrist		3.7 (↓69%)	43 (↓39%)
Right median	Finger I		1.3 (↓97%)	37 (↓35%)
	Wrist		2.7 (↓78%)	42 (↓40%)
Right ulnar	Finger V		1.8 (↓89%)	36 (↓39%)
	Wrist		2.0 (↓78%)	45 (↓33%)
Left fibular	Toe I			Absent
	Ankle			Absent
Left posterior tibial	Toe I			Absent
Right fibular	Toe I			Absent
	Ankle			Absent
Right posterior tibial	Toe I			Absent
Motor				
Left median	Wrist	4.2 (↑40%)	1.7 (↓92%)	
	Elbow		1.5 (↓91%)	40 (↓38%)
Right median	Wrist	4.0 (↑33%)	7.7 (↓65%)	
	Elbow		5.0 (↓72%)	33 (↓49%)
Right ulnar	Wrist	2.7 (Normal)	8.9 (Normal)	
	Elbow		7.7 (Normal)	39 (↓39%)
Left fibular	Ankle	Absent		
	Fibular head			Absent
Left posterior tibial	Ankle	4.5 (Normal)	0.3 (↓98%)	
Right fibular	Ankle	Absent		
	Fibular head			Absent
Right posterior tibial	Ankle	Absent		

**Figure 4:**
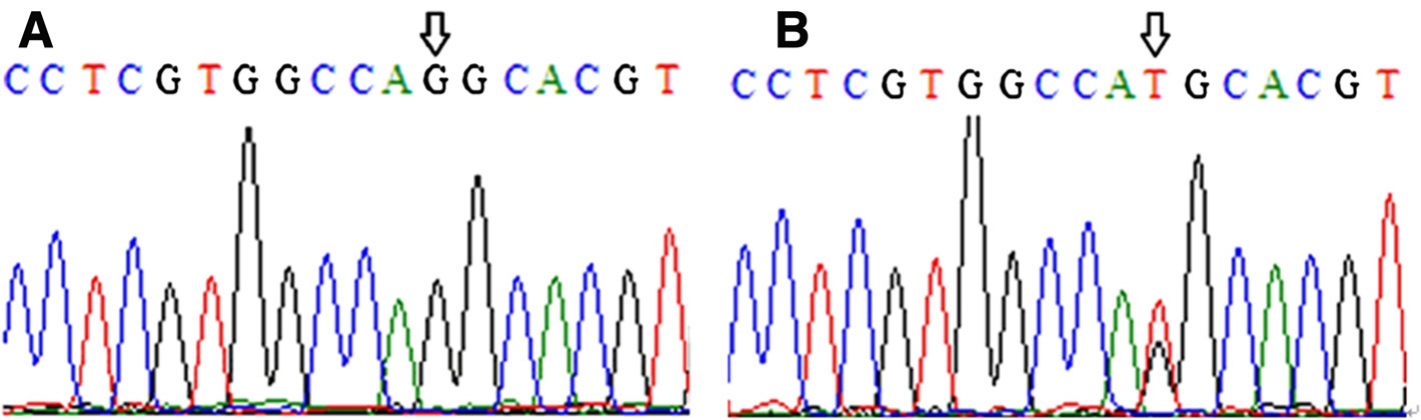
**Chromatograms of a Cx32 mutation.** The arrow in chromatogram **A** represents the mutation site (T to G transition at nucleotide 278) in the male Chinese patient with X-linked Charcot-Marie-Tooth disease. The arrow in chromatogram **B** represents the site of a T278G heterozygous mutation in the patient’s mother.

Genetic testing of this patient showed a T to G transition at nucleotide 278 in connexin 32, resulting in changing a methionine to a arginine. His mother had T278G heterozygous mutation (Figure 4). His father had normal sequence.

The patient had normal strength in all muscle groups, but the deep tendon reflexes were diminished bilaterally when he was in our department. After hospital discharge, there was no longer onset of his neurological symptoms.

## Conclusion

The patient in this case had hyperpyrexia two weeks before onset of the disease. The clinical manifestations showed repeated transient weakness of the limbs, slurred speech and dysphagia. Neurologic examination revealed muscular atrophy of distal limbs, diminished deep tendon reflexes in all extremities and giving the characteristic inverted champagne bottle appearance. Brain MRI scans presented white matter abnormalities in the internal capsule, corpus callosum and periventricular areas. Two months later,his third brain MRI showed that the signal abnormalities had almost disappeared. Electrophysiology certified peripheral neuropathy. The genetic analysis showed a pathogenic mutation in GJB1. This patient was diagnosed as CMTX1 accompanied by reversibility transient white matter abnormalities.

CMT is classified as autosomal dominant, autosomal recessive or X-linked CMT (CMTX). CMTX is divided into a dominant CMTX1 and four recessive CMTX 2–5 [3]. At present we think that CMTX1 is caused by mutation in the GJB1 gene on chromosome Xq13.1 at codes for the connexin 32 protein (Cx32). Cx32 is expressed in myelinating Schwann cells and localized to paranodal regions and Schmidt Lanternmann incisures. Six connexins oligomerize to form hemichannels or connexons. When properly opposed to each other on cell membranes, two connexons form gap junction channels that permit the diffusion of ions and small molecules [4]. The diffusion of ions and small molecules may be restricted by Cx32 mutation, which causes the defect of nervous functions.

Case reports show that transient cerebral white matter lesions of CMXT1 can be acute or subacute ataxia, dysarthria, hypoesthesia, aphasia, hemiplegia or quadriplegia etc., which can be alleviated in several hours or several days, or maybe attack repeatedly. Usually the CMXT1 symptoms are severe in male patients, but asymptomatic or mild in female carriers [5]. Although there are reports of some female heterozygotes who have serious manifestations [6].

Predisposing factors to transient cerebral white matter lesions of CMXT1 may be return to plain from high altitude area, fever, infection, can even be no clear reason [4],[7]–[10]. The CMTX1 typical MRI shows abnormalities in non-enhanced, confluent, symmetrical corpus callosum and periventricular white matter [5]. These episodes may be related to temporary myelin vacuolation as suggested by restricted diffusion in the affected areas on MRI. Using transgenic mice shows that the mutation of Cx32 loses its function in Schwann cells and oligodendrocytes, resulting in myelination defects of the peripheral and central nervous system [11]. Dysfunctional gap junctions as a result of the mutation enables these cells have no ability to adjust fluid exchange, these could explain the restricted diffusion on MRI [9]. Follow-ups and literature reports about cerebral white matter lesions present that brain MRI can be fully recovered or improved in hours, days or months.

This paper reports the electrophysiological examination of the patient, which presents that the peripheral nerve axon and myelin sheath are involved. It is also reported previously that axon and myelin sheath can be involved at the same time [6], or the axon is involved mainly [12].

The male patient with novel T278G mutation of GJB1 had hemizygous, and his mother had heterozygous mutation. His mother, his mother’s mother and his mother’s sister presented pes cavus. His father had normal sequence. The CMTX1 has been known to be an X-linked dominant inheritance pattern. In CMTX, male patients tend to be severely affected, and females are generally asymptomatic [6]. The GJBl mutation in the male patient results in the change of met93arg. We speculate that the mutation may lead to loss of functional gap junction protein in Schwann cells and oligodendrocytes, which causes the dysfunction in central nervous system.

We reported a T278G mutation causing a methionine to arginine substitution on code 93 (M93A). Bell et al. [13] presented a A277G mutation on code 93 (M93V). Both mutations affected the same amino acid (methionine). The clinical manifestations of M93V included tremor, spasticity, brisk reflexes and extensor plantar responses. On MRI there was atrophy of the cerebral cortex and cerebellum. While the phenotype of M93A presented transient and recurrent weakness of the limbs and diminished deep tendon reflexes in all extremities. MRI showed reversibility white matter lesions in the internal capsule, corpus callosum and periventricular areas.

In conclusion, T278G is a new mutation in the GJBl gene, which may be associated with CMTX1. The disease shows paroxysmal leukoencephalopathy. Axonal and myelin lesions result in weakness of limbs, slurred speech, swallowing difficulties. Reversible cerebral white matter damages of corpus callosum and periventricular areas are the characteristics of the disease on MRI. For patients who have peripheral nerve damages and reversible cerebral white matter degeneration, CMTX1 should be considered.

## Consent

Written informed consent was obtained from the patient’s parent for publication of this Case report and any accompanying images. A copy of the written consent is available for review by the Editor of this journal.

## Abbreviations

CMTX1: X-linked Charcot-Marie-Tooth disease type 1

MRI: Magnetic resonance imaging

GJB1: Gap junction protein beta l

PCR: Polymerase Chain Reaction

CNS: Central nervous system

Cx32: Connxin 32 protein

BAEP: Brain-stem auditory evoked potential

## Competing interests

The authors declare that they have no competing interests.

## Authors’ contributions

YZ, YX, HW, YL and JL diagnosed, treated and followed up the patient, YX and XZ made a contribution to laboratory work, YZ drafted the first manuscript, YX revised the manuscript. All authors read and approved the final manuscript.
